# Contact lens embedded holographic pointer

**DOI:** 10.1038/s41598-023-33420-8

**Published:** 2023-04-27

**Authors:** François-Maël Robert, Bernard Abiven, Maïna Sinou, Kevin Heggarty, Laure Adam, Vincent Nourrit, Jean-Louis de Bougrenet de la Tocnaye

**Affiliations:** 1 Département Optique, IMT Atlantique, Technopôle Brest-Iroise, 655 Avenue du Technopôle, CS 83818 – 29238, Brest Cedex 3, France; 2 LCS, 14 Place Gardin, 14000 Caen, France

**Keywords:** Imaging and sensing, Micro-optics

## Abstract

In this paper we present an infrared laser pointer, consisting of a vertical-cavity surface-emitting laser (VCSEL) and a diffractive optical element (DOE), encapsulated into a scleral contact lens (SCL). The VCSEL is powered remotely by inductive coupling from a primary antenna embedded into an eyewear frame. The DOE is used either to collimate the laser beam or to project a pattern image at a chosen distance in front of the eye. We detail the different SCL constitutive blocks, how they are manufactured and assembled. We particularly emphasize the various technological challenges related to their encapsulation in the reduced volume of the SCL, while keeping the pupil free. Finally, we describe how the laser pointer operates, what are its performances (e.g. collimation, image formation) and how it can be used efficiently in various application fields such as visual assistance and augmented reality.

## Introduction

Among Brain-Computer-Interfaces (BCI)^1^, eye trackers have become among a popular interface to assess and modulate sensorimotor and cognitive functions. They have been used to achieve basic tasks such as selection, manipulation, navigation^2,3^. The analysis of eye tracking data showed that eye movements could also provide important information on cognitive processes (e.g. fatigue, mental workload, etc.^4^), suggesting that eye tracking could provide an alternative or a complementary signal to current BCI applications^5^. For instance, in future augmented reality systems, eyes will become a common key user interface, replacing standards like cursors, touch-screens, touch-pads or keyboards to convey visual intents or commands and to identify cognitive loads. Therefore, merging visual attention with designation tasks is of great interest for many applications. Performing a visual designation can reduce the operator workload, allowing them to concentrate on their main mission while establishing a new link between planning, control functionalities, and sensory coordination. In parallel, recent advances in microelectronics and nanofabrication on flexible substrates have enabled sensors, circuits, and other essential components to be integrated into contact lenses^6,7^. For instance, we have recently demonstrated how a contact lens embedding one or two VCSELs could be useful to facilitate eye tracking, particularly where an eye tracker has to be integrated into a constrained environment (e.g. VR or AR HUD^8,9^, binocular loupes, etc.). However, the commercial VCSELs we used did not have a significant beam divergence and could not be used to project a precise pattern at a distance from the eyes greater than a few centimeters. In addition, due to the small emitted power required to comply with ocular safety regulation rules, the system was not usable in practice with sensors far from the eye. This is the limitation our paper intends to bypass by having a device making it possible to project, from the eye, a point or a pattern at several tens of centimeters. This would open up new applications in human machine interactions and more specifically BCI.

We present here how the introduction of a Diffractive Optical Element (DOE) within the scleral contact lens (SCL), in front of the VCSEL, can be used to collimate the laser beam or to project an image at a chosen distance. We detail how this optics is made, aligned and mounted on the laser before being encapsulated into the SCL. The contact lenses we used are scleral lenses which provide several advantages over standard contact lenses: they are stable on the eye, they are not in contact with the cornea and offer more volume to encapsulate components^10^. The paper is organized as follow: we first present the results obtained with the final SCL prototype (pattern projection, collimation, detection etc.) before discussing them in the Discussion section. The design, manufacturing, and assembly of the various SCL building blocks are presented at the end in the section Material and methods.

**Figure 1:**
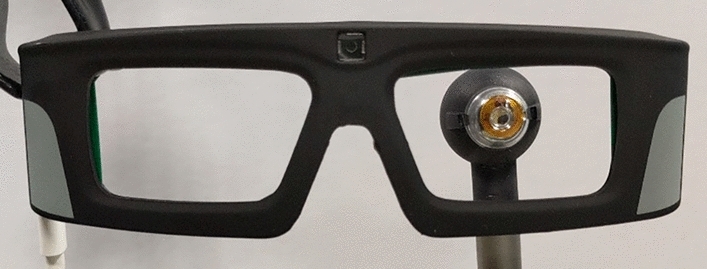
Eyewear and SCL mounted on a mock-up eye.

The basic eye-tracking configuration has been described in detail elsewhere^11^. It associates an electronic SCL powered by inductive coupling and an eyewear with the primary antenna (Fig. 1). The lens is made in PMMA and has a 16.5 mm diameter. It embeds a flexible secondary antenna and electronic circuits including one VCSEL (at 850 nm). The eyewear can be equipped with additional sensors (cameras or position sensitive devices (PSD)^11^) to detects the VCSEL beam spot and follow the eye motions^12^.

In order to control the VCSEL’s beam divergence, a DOE is then placed in front of the VCSEL. Two DOEs have been manufactured and tested, one to reshape the laser beam into a cross pattern, the other to collimate the beam. Figure 1 shows the final SCL encapsulating all the above-mentioned functions and its driving eyewear.

## Preliminary results before encapsulation

**Figure 2:**
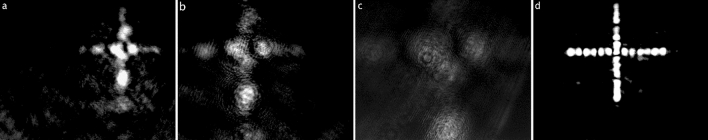
The use of a DOE inside the SCL allows to project a cross on the CMOS sensor at a distance ((**a**) 7.5 cm, (**b**) 12 cm, (**c**) 20 cm). (**d**) Test outside of the SCL. Better image quality can be obtained on the CMOS sensor at 7,5 cm if the VCSEL beam illuminates a larger area of the DOE.

A preliminary experiment has been carried out to scale and test the quality of the image formation and collimation at different distances before assembling and encapsulating the SCL (Fig. 2). In this experiment, a VCSEL, powered by a regulated power supply, illuminates a DOE etched on a 1 mm-thick glass plate. A CMOS sensor is placed after the DOE. The distance between the VCSEL and the DOE (z-axis) is critical for a correct image formation (cf. Fig. 2) or collimation. The diffractive design has been computed for a focal distance of 800 µm. Due to this short focal length, the tolerance on the z-position between the VCSEL and its distant conjugated image through the DOE is also very small (40 µm). The x- and y-directions are less critical. When the DOE moves in the (xy) plane, the image moves accordingly in the same direction with just a decrease in the optical power concentration. The tolerance along x and y directions is estimated to 240 µm (if the misalignment between the VCSEL and DOE is larger, then the illuminated surface of the DOE will be too small to produce the desired effect).

### Image formation

Regarding the use of a DOE for image formation, Fig. 2 illustrates the image formation of a cross when the CMOS sensor is placed at different distances. The DOE was calculated to form a cross at infinity but, as depicted the cross can already be observed at short distances. The image slightly increases in size with distance because the collimation of the laser is never perfect but is contained in a 3° field of view. At 7.5 cm the cross is 3.5 mm large and 0.7 mm thick.

The reduced quality of the diffractive pattern is due to the relatively small illuminated part of the DOE. Currently the DOE size is 225 × 225 µm. Its resolution is 0.75 μm and the illuminated area has a diameter of 95 µm. Keeping the same DOE resolution but illuminating a larger area (224 µm), for instance by folding the optical path within the lens to increase the optical distance between the VCSEL and the DOE (1600 µm), would give better results as depicted in Fig. 2d.

### Collimation

**Figure 3:**
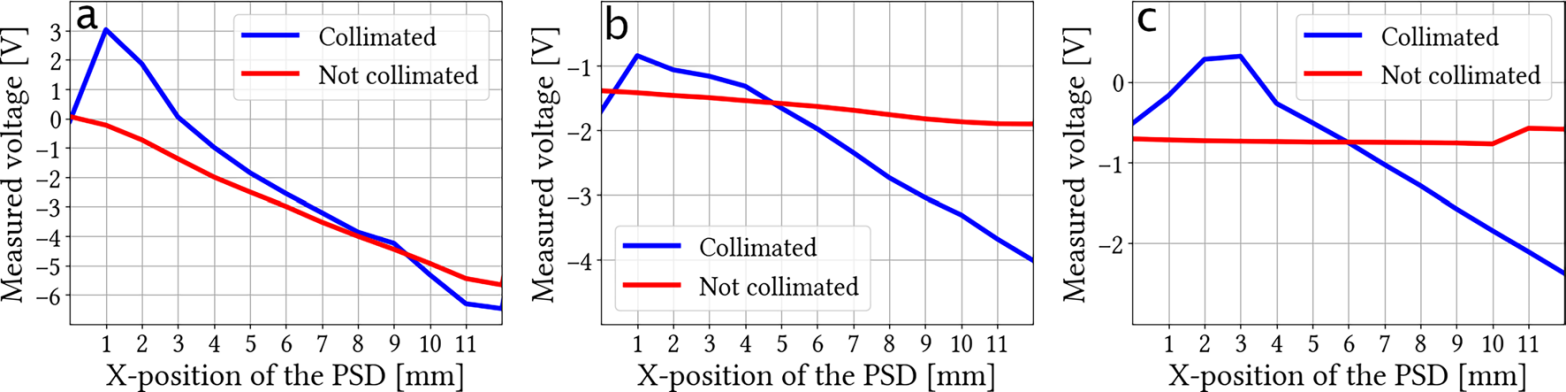
PSD response (in blue for the collimated VCSEL in red for the non-collimated one) as a function of spot position for different distances from the PSD: (**a**) 8.5 cm, (**b**) 20 cm, (**c**) 40 cm. If the VCSEL post size on the PSD is too large (**b**,**c**) then the PSD cannot accurately measure the spot position.

The same experiment was then carried out with the collimating DOE. The VCSEL light goes through the diffractive element and is collimated. After collimation, the measured divergence angle of the laser beam is less than 1.8°, which is more than four time smaller than the initial VCSEL divergence according to its datasheet (8°). The advantage of collimating the beam with respect to shaping it, is to facilitate its detection by a position sensitive detector (PSD). We thus compared the VCSEL beam detection, with and without DOE, for different distances to a PSD. The PSD used here was a Hamamatsu S1880, associated with the control circuit Hamamatsu C4674-01. For each position, the average of 8000 voltage values over one second was retained. Previous work proved however that only 50 samples could be kept without loss of precision^11^, allowing to use the PSD for spot detection in real-time (at 200 Hz). The distances tested for the PSD were 8.5 cm, 20 cm, and 40 cm. Results are presented in Fig. 3. The 40 cm distance corresponds to the case of a user pointing an object on a screen or a laptop with his eyes. Results show that in the first case (Fig. 3a), the collimation has no significant effect on the resolution of the PSD but that when the distance increases, it becomes, on the contrary, essential, to be able to continue to detect accurately the laser beam.

## Results

**Figure 4:**
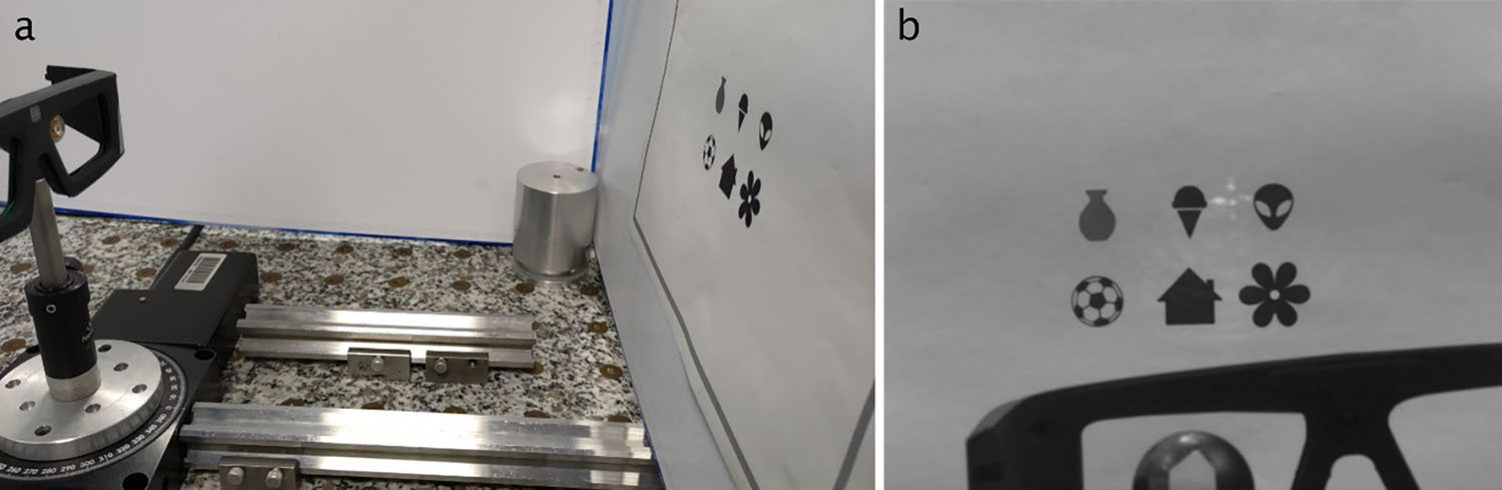
(**a**) Optical set-up used to validate the device. (**b**) The cross projected from the SCL by the laser pointer is clearly visible on the screen when imaged with an infrared camera.

The second experiment has been carried out with a SCL encapsulating the whole functions (illumination and beam shaping). We chose to encapsulate an imaging DOE because it represents a more demanding case than collimation. The optical set-up used to validate the results is made up of a mock-up eye, on which the SCL is fixed. It is powered by the primary antenna in the eyewear, placed at 13 mm from the SCL. A screen depicting various symbols is placed in front of the SCL, at 30 cm (Fig. 4). The SCL projects a cross on the screen as shown Fig. 2, showing that the encapsulation process does not distort the pattern created by the DOE. The mock-up eye is mounted on a rotative plate, allowing to move the cross pattern to a specific icon, thus demonstrating the potential of the SCL for target designation. A video showing the cross motion following the eye movements is attached as supplementary material to this paper.

## Discussion

As presented in the introduction, eye tracking presents a strong potential for a number of applications but it also requires a precise calibration which is not always easy to achieve and maintaining good tracking is difficult^13^ due to different factors such as head movements, changing illumination, calibration decay, etc. In order to bring back the measurement at the heart of the problem (similar to early recordings of eye movements using a pointer directly connected to the eye^14^), we had initially built a first prototype with just a VCSEL and no DOE^10^. With this first prototype, the beam diverged too quickly and after a few tens of centimeters the beam spot was too large and the illumination too low to effectively point where the user was looking at. The VCSELs can then be used as markers to track eye movements. This presents a number of advantages when compared to standard video-oculography (e.g. it is easier to track a spot rather than the pupil) but still relies on a valid calibration.

The device presented here is the first true visual pointer as the CL allows to directly position, thanks to a luminous point, the gaze position. In addition, it also makes it easier to use PSD to detect the beam direction.

In terms of manufacturing and integration, this is, to our knowledge, the first study reporting the integration of a DOE into a contact lens.

Beam shaping is a key element in moving from a lens with a simple embedded light source to a true laser pointer. However, due to the limited volume available designing, manufacturing, and embedding within the contact lens a beam shaping element is not trivial. In this paper, we present technical details on the different solutions studied and retained that allowed us to manufacture a 1,44 mm^2^ DOE, place it at 680 µm from the VCSEL with a positioning tolerance of 40 µm and encapsulate it successfully into a contact lens.

The imaging quality of our system is related to the illuminated DOE area. Improving the projected pattern quality assumes thus an increase in the DOE lightened surface, which is limited here by the VCSEL divergence (8°) and the necessity to limit the lens thickness. A solution could be to use multiple beam reflections through a light guide, as presented in^15^. A related key issue is the assembling robustness of several optical elements together with small tolerancing and to guarantee their rigidity. Several gluing techniques have been tested, none are really compatible with a manufacturing process. This point should be investigated further with respect to the encapsulation constraints into rigid SCLs and according to the future forces on them.

In terms of applications, the demonstrated collimation of the laser beam allows a better flexibility of use of the PSD solution (e.g. when compared to^11^), in particular by relaxing the choice of its position which can be located up to few tens of centimetres away from the SCL. Moreover, collimation improves pointing accuracy which confirms the possibility of using such a pointer, as an actuator or visual pointer, for instance, when combined with a blink command as shown in^16^. Alternatively, the DOE can be used to create a pattern, an image, or an icon at a given distance, which can be detected by a camera without disturbing the wearer because it is not visible to him. This pattern can be recognized, for instance, to lock-up or designate targets^17^. It could be useful for instance, for some supervision or tutorial applications (e.g. visual assistance), to materialize precisely where the wearer is looking at.

An alternative method to materialize the point of fixation could be to use a standard eye tracker to control a motorized laser pointer placed, for instance, on spectacles. We have built such a system in a separate study^18^. There are however three issues with such approach that are avoided with the CL pointer. First, a motorized laser pointer relies on the performances of a standard eye tracker with all the limitations listed above (calibration decay, etc.). Second, there is inevitably some latency between the eye movements and the laser movement. Third, accurate and fast pointing requires a quality of motorization which can be too heavy for an embedded system.

In terms of safety, the prototype presented in this study has not been tested on a human eye because the presence of the DOE takes the lens beyond the limits of what can be acceptable in terms of central thickness (1700 μm). This is why we suggest using a waveguide to fold the optical path and reduce the lens thickness. If this thickness can be reduced then the lens could be easily wearable since the electronics is entirely inside the lens. The presence of electronic circuitry within the lens also implies a reduced oxygen permeability which limits the time of use of these lenses but this could easily be improved by first using a material with a better Dk than PMMA. Furthermore, preliminary tests on rabbits and toxicological analysis (ISO 10993) as part of a current EC marking procedure showed that the lens could be used safely for at least 30 min. In terms of functioning, the emitted power (120 µW at 850 nm) is too low to impact the cornea, even in the worst-case scenario (NF EN 60825) and the heating of the VCSEL at the surface of the lens is low as well (< 0.5 °C).

A direct extension of this optical configuration consists in directing the beam towards the retina instead of in front of the viewer. This could be achieved by folding the beam and using a waveguide as proposed in^15^. Although, it assumes the use of visible VCSEL^19^ (to stimulate the retina) which only begins to be commercially available, such a device represents only a modest improvement over the current SCL and could be the first basis for a system of command and alert for future man–machine interactions.

## Materials and methods

This part details the fabrication and assembly of the various SCL building blocks before their encapsulation.

### Electronics and RF circuits

**Figure 5:**
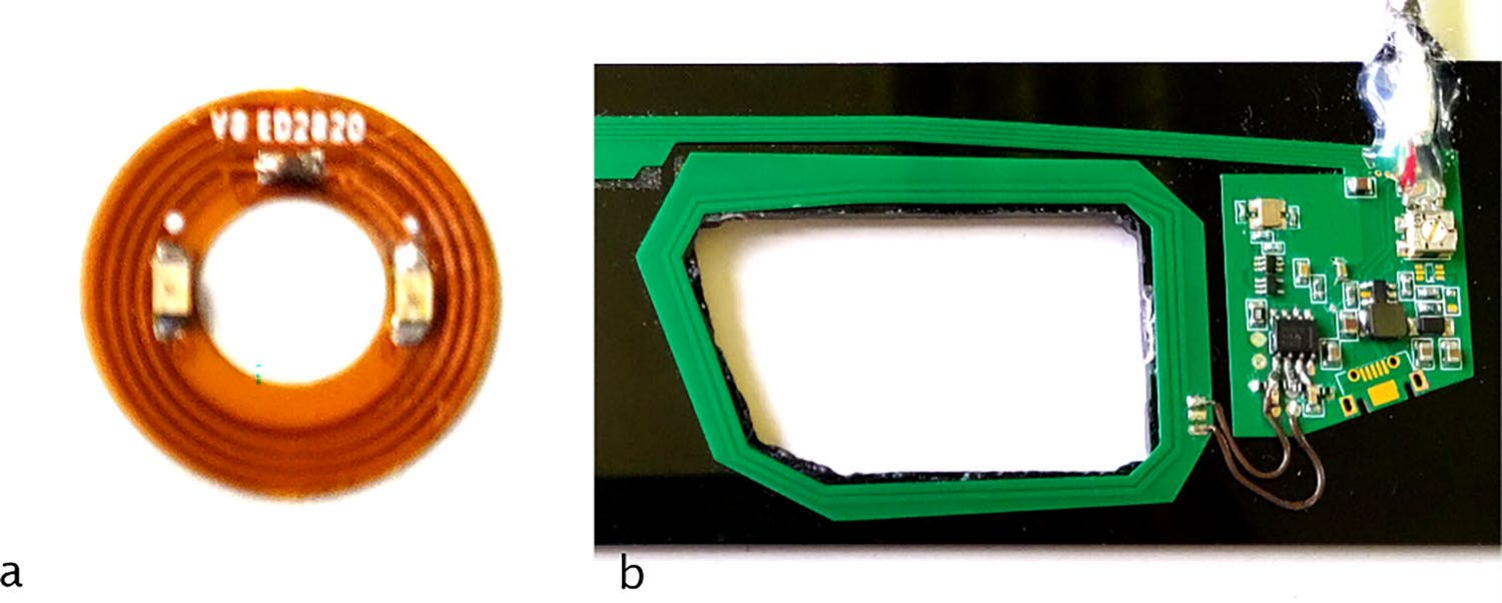
(**a**) the electronics encapsulated into the SCL with the coils, two VCSELs and the according capacitor, (**b**) the electronics inside the eyewear, with the primary antenna coil on the left and the transformer on the right.

Our laser pointer is made of two main elements, the first one is the SCL, the second is the eyewear. The SCLs used are rigid scleral contact lenses guaranteeing the stability on the eye, no direct contact with the cornea and offering a larger volume than the soft contact lenses. A key parameter of our design is that the pupil area is kept free of any element. The electronics and power harvesting elements encapsulated into the SCL consist of a double-sided flexible ring of 4.8 mm inner diameter and 10.5 mm outer diameter. It contains one infrared VCSEL (emitting at 850 nm), powered by induction, and a coupling capacitor (Fig. 5a). The electrical power circulating in the primary coil is 340 mW and the power emitted by the VCSEL is 120 µW. The spacer and the DOE are mounted on the VCSEL as described, here after in section “Spacer design”. Regarding the eyewear (Fig. 5b), it includes the primary coil which is used to power and trigger the SCL. This part is described extensively in^10^ and could also include detection devices such as cameras or PSD arrays^11^.

### DOE design, fabrication and cutting

**Figure 6:**
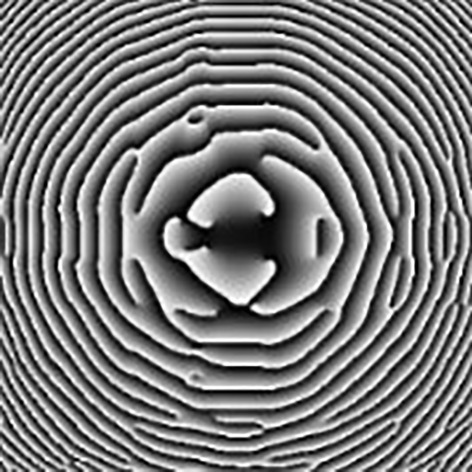
Phase pattern of the DOE used to correct the VCSEL beam divergence and form a cross in the far field.

As explained above, the DOEs are used either to collimate the VCSEL beam, when using for instance a PSD sensor placed at a given distance from the eye^11^ or to project a pattern (e.g. here a cross) at a given distance in front of the eye, in the gaze direction. The DOEs are designed using a modified conventional three-stage Iterative Fourier Transform Algorithm (IFTA)^20,21^. The DOEs are multi-phase level elements, etched into a layer (thickness ~ 1.8 µm) of spin-coated S1813 photoresist (MicroChem) on 175 µm thick borosilicate glass substrates using a custom-built, massively parallel-write photoplotter^22,23^. Typical DOE (Fig. 6) experimental diffraction efficiencies of 70–75% are generally observed. The usable DOE size is determined here by the VCSEL beam divergence and the VCSEL to DOE distance which encapsulation constraints limit to be less than 1 mm. Therefore, considering our VCSEL divergence (8°) and a distance to the DOE of 680 µm, (to limit the CLP’s total thickness), the usable surface is around 0.3 mm^2^, as the spot’s diameter is 95 µm. Here, the size of the DOE is of 225 × 225 µm.

**Figure 7:**
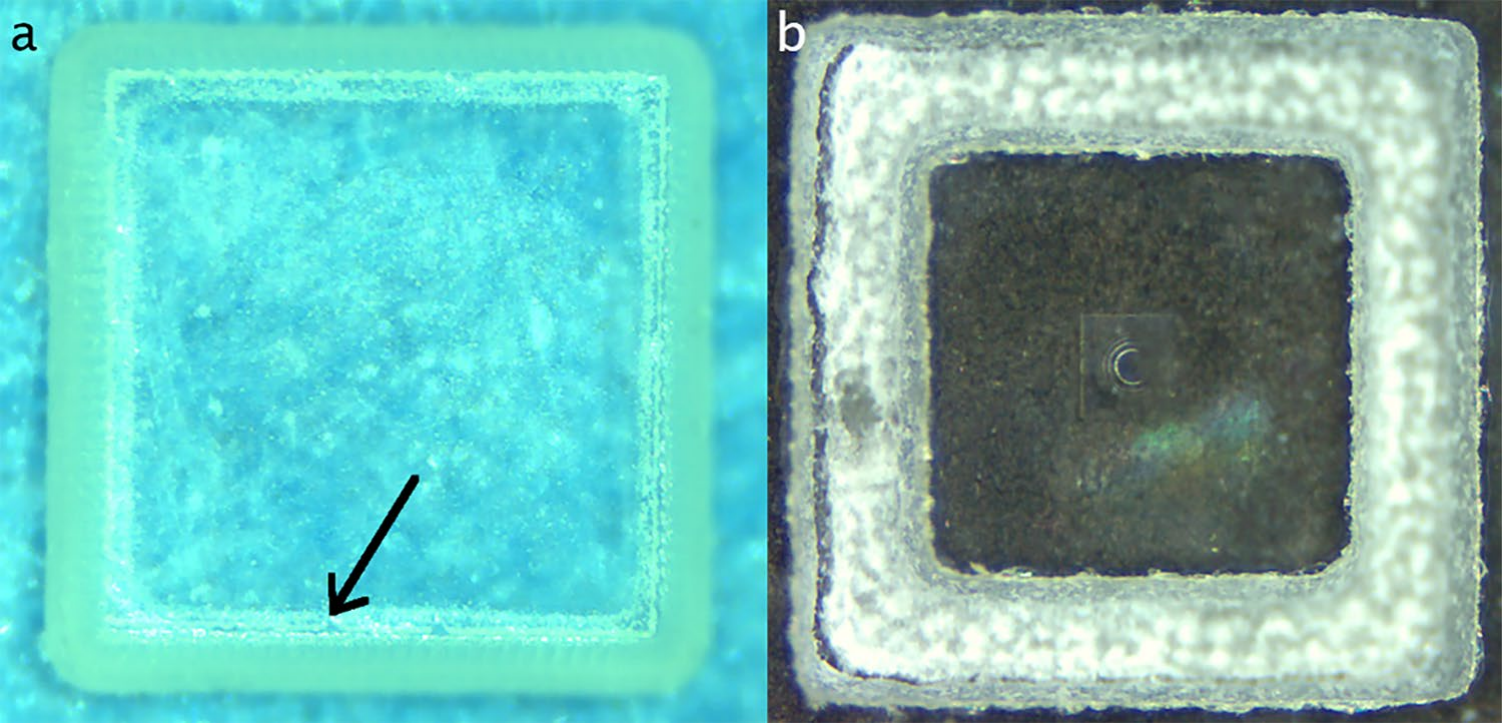
DOE of dimensions 225 × 225 µm on a glass square of 1.2 × 1.2 mm (**a**) with laser ablation. The black arrow points to the Ormocomp area damaged by heating during the cutting process. (B) With sandblasting.

Before encapsulation DOEs are cut into small squares (around 1 mm^2^). Different cutting techniques have been tested. A first trial has been made by using a 1064 nm laser (from the company Laser Cheval). An issue with laser ablation is related to the glass heating during the process that can damage the DOE. It is particularly the case when using photoresist. The consequence is the need for introducing a dead zone between the DOE and the substrate edge which increases the overall element size and hence the encapsulation constraints. In that case, the smallest size we achieved to cut was a 1.2 × 1.2 mm^2^. The parameters are 50 ns pulse duration, 20 kHz repetition rate, 20 W average power, 10 mm/s scan speed, and 15 passes. They have been fixed after various experiments, to find the rights values that allow to cut the glass without burning the photoresist layer deposited on it. Laser ablation has also been tried with a femtosecond laser 20 W, also from the company Laser Cheval. In that case, the photoresist is replaced by Ormocomp, to reduce the sensitivity of the layer to heating. Ormocomp is a hybrid organic–inorganic polymer (produced by the company Microresist) with excellent optical transmission and mechanical properties. The result is presented in Fig. 7a. We can notice that the edges are very clean and sharp. Ormocomp is burned by the laser heating only around 50 µm on the edges of the square. The use of Ormocomp then would allow to reduce the size of the cut square to 1 × 1 mm. Another solution has been tested using sandblasting provided by Icoflex in Switzerland, also with an Ormocomp layer. An example is presented in Fig. 7b. The edge is less sharp and the size of the blasted part depends on the glass substrate thickness, therefore reducing the usable area. However, approximately two months after the cut, the DOE peeled off, probably due to the constraints. We thus did not continue to use that method.

### Spacer design, manufacturing and assembling

**Figure 8:**
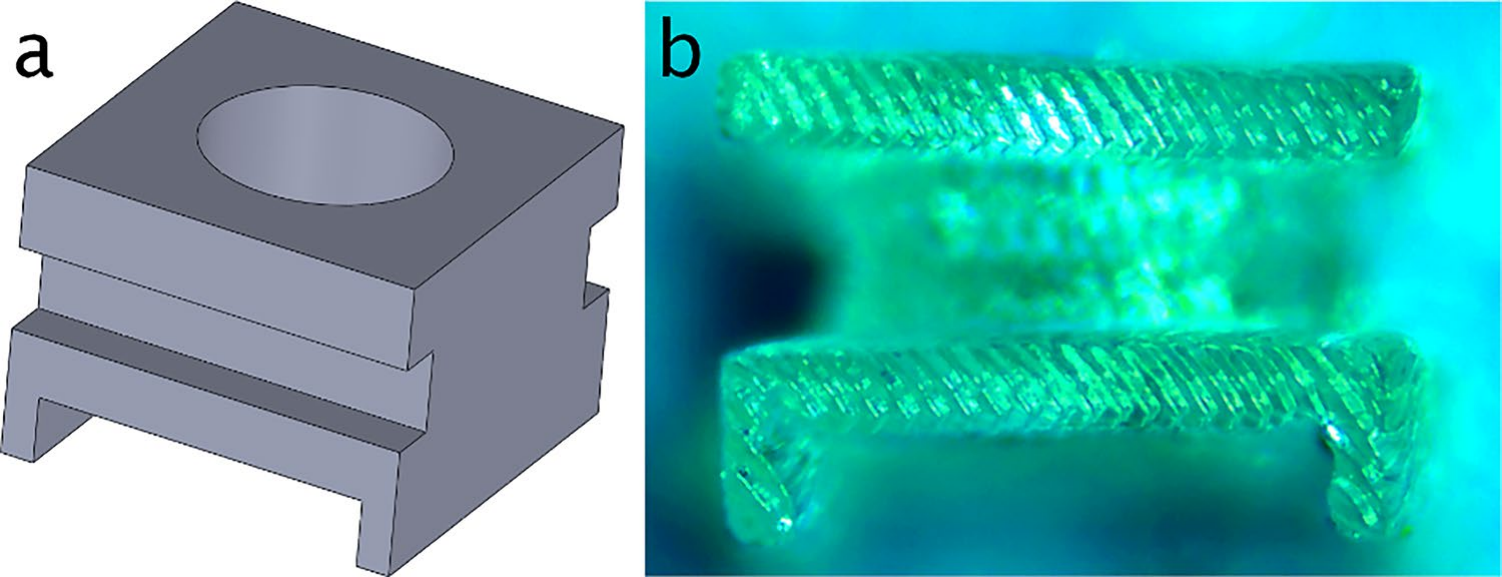
(**a**) 3D view of the spacer, (**b**) photo of the printed spacer.

This is another critical part of the process. First, due to the very small focal length required for illuminating the DOE (800 µm), either to collimate the laser beam or to image a pattern at a finite given distance in front of the eye, the positioning tolerance is small (~ 40 µm) and must be adjusted accurately. Second, in order to be sure that this distance is not modified during the assembling and encapsulation process, it is necessary to bind together the VCSEL and the DOE. For this purpose, we have designed and manufactured a specific spacer. This spacer (Fig. 8a) was designed using Solidworks software, and fabricated by resin 4 K 3D printing (Fig. 8b). We used a flexible photopolymer resin to print the spacer. This allows us to precisely set the DOE at the correct distance from the VCSEL, thus adjusting for various parameters (e.g. the VCSEL cover height can vary, etc.). We designed and tested an accordion type structure to facilitate the material deformation (Fig. 8a). The distance between the top of the housing of the VCSEL and the DOE’s plane is around 680 µm. The spacer has a height of 680 µm, with disparities due to the accuracy of the 3D printer. The part has fixing feet on the bottom to maintain it on top of the VCSEL housing, and a plate on the top on which is placed the glass substrate containing the etched DOE. The top surface is a square of size 1.2 × 1.2 mm, corresponding to the DOE dimensions. The spacer has a cylindrical hole in its middle, to free the light path from the VCSEL. The use of a flexible spacer allows to obtain the required VCSEL-DOE distance, by putting the assembly in compression until the projected pattern is as desired. Then, when the distance is fixed, another spacer in solid resin is printed, to fix definitively the distance during all the mounting and encapsulation process.

A fine adjustment of the focus can be obtained dynamically by using a visual optical control as detailed in the next section.

### Assembling before encapsulation and gluing

**Figure 9:**
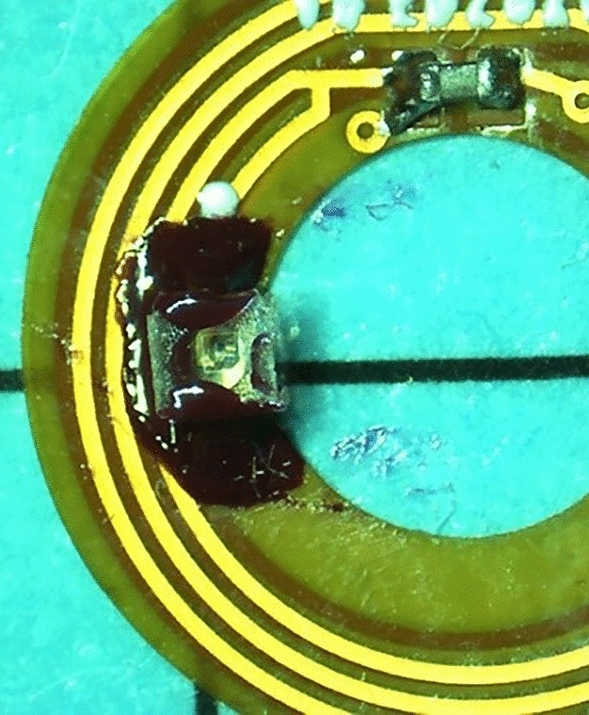
Circuit with a DOE mounted on the left VCSEL.

The final critical point before encapsulating the circuit is the sealing of the VCSEL, spacer and DOE assembly. We tested various glues which were either too fluid (optical UV glue Norland Optical Adhesive 65 or UVS 91 UV glue) so that when deposited with a needle, the glue drop spread and filled the central hole of the spacer, or with a too high viscosity (SU8 glue) so that it was impossible to drop a little drip on the spacer. Finally, the most appropriate solution was a small drop of nail polish made with nitrocellulose (Rimmel 60 Seconds Super Shine, as used in^24^) on each longitudinal extremity of the spacer. The nail polish presents a good viscosity and dries without exposition to UV, which is more handful for our purpose. After few minutes to let the polish dry, another drop was deposited on each lateral extremity of the plate surface, to provide a slight adherence. Then the DOE was placed on top of the spacer, and the same operation was repeated. A drop of nail polish was deposited on each side of the spacer to glue the glass substrate. At this stage, pressure was applied on the structure to finely adjust the focus and obtain the best collimation or pattern imaging (as shown in Fig. 9). At each step, the propagation of the light through the structure was verified. An IR camera was used to finally check that the correct pattern is projected when the light goes through the DOE.

### Circuits and DOE encapsulation

**Figure 10:**
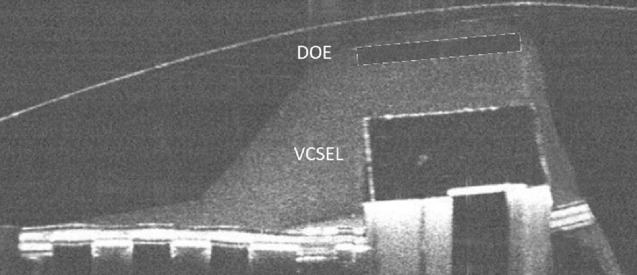
OCT view of the circuit inside the contact lens with a schematic representation of the DOE.

Once the DOE is fixed to the VCSEL, the whole electronics is then sandwiched between 2 pre-manufactured pucks which are sealed together before the upper and lower surfaces are lathed to manufacture the lens curvatures and to obtain the final wearable contact lens. During pre-manufacturing, a hollow ring is initially etched in the upper surface of the bottom puck to accommodate the optical system (VCSEL + spacer + DOE) Fig. 10.

**Figure 11:**
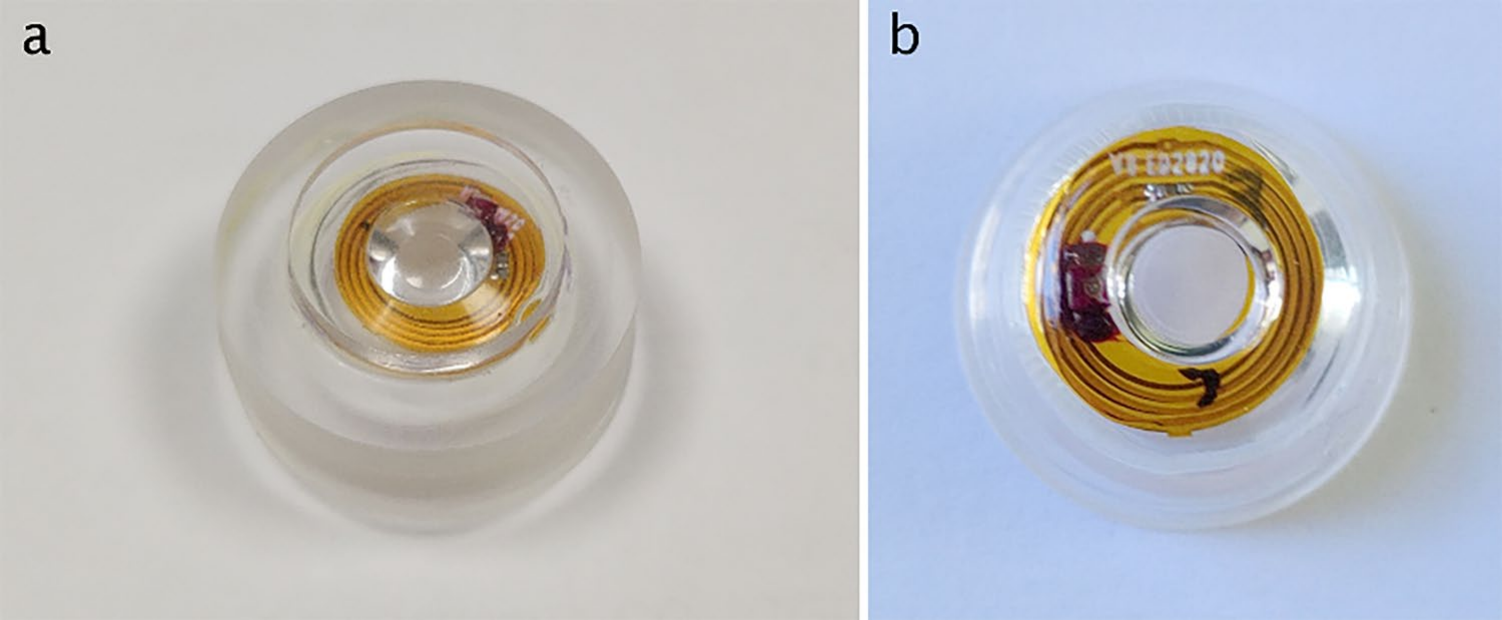
(**a**) View of the SCL pucks before lathing, and (**b**) of the final lathed SCL.

To assemble the two pucks, UV-glue (Loctite AA 3301) is deposited with a needle on the edge of the bottom part, then the top part is put in compression with the press. During the compression, the glue is UV cured. The whole assembly forms a new puck, with the circuit encapsulated inside. This puck is finally formed into a SCL shape with a lathe, as depicted in Fig. 11.

**Figure 12:**
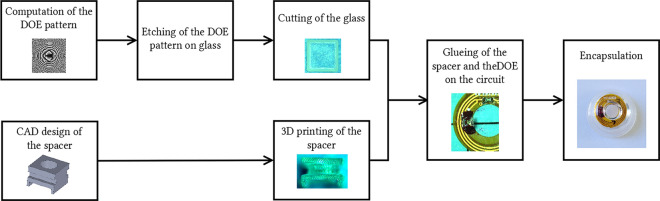
Diagram representing the main steps for the manufacturing of the contact lens embedded holographic pointer.

The overall process to manufacture this contact lens laser pointer is depicted in Fig. 12.

## Supplementary Information

Supplementary Video 1.

Supplementary Video Legend.

## Data Availability

All data generated or analysed during this study are included in this published article. The raw datasets for Fig. 3 is available from the corresponding author on request.
